# Associated factors in distinguishing patients with brucellosis from suspected cases

**DOI:** 10.1186/s12879-019-4662-3

**Published:** 2019-12-09

**Authors:** Jingjing Luo, Huixin Yang, Fangfang Hu, Siwen Zhang, Taijun Wang, Qian Zhao, Ruize Wang, Qing Zhen

**Affiliations:** Department of Epidemiology and Biostatistics, Key Laboratory of Zoonosis Research, Ministry of Education, Jilin University School of Public Health, No.1163, Xinmin Street, Chaoyang District, Changchun, Jilin 130021 People’s Republic of China

**Keywords:** Brucellosis, Suspected case, Confirmed case

## Abstract

**Background:**

To investigate the risk factors for brucellosis in suspected cases of the disease.

**Methods:**

A self-designed questionnaire was developed to collect data from 3557 people whose initial visit site was the Songyuan Center for Disease Control and Prevention (CDC) from January 1st, 2009 to December 31st, 2012. After collecting blood samples, a plate agglutination test (PAT) and serum agglutination test (SAT) were used to distinguish the patients with brucellosis from the suspected cases.

**Results:**

Sex, occupation (farmers and herdsmen), contact with abortion products, and contact with feces were the main risk factors for brucellosis in the suspected cases (all *P* < 0.05). No difference existed between the confirmed cases and suspected cases in the demographic characteristics, contact with animals (except swine), contact with substances, or clinical symptoms (except fever). However, the confirmed cases showed significant differences from people without brucellosis in demographic characteristics, contact with animals (except cattle and swine), contact with substances, and clinical symptoms. Suspected cases exhibited significant differences from people without brucellosis in the demographic characteristics (except education), contact with animals (except swine), contact with substances (except dust), and clinical symptoms (except chills and acratia). *Brucella* was cultured from the blood samples of three of 30 suspected cases with fever. Using AMOS-PCR and agarose electrophoresis, the detailed species of *Brucella* strain was identified as *Brucella melitensis*.

**Conclusions:**

Abortion products and feces are the main risk factors for brucellosis in suspected cases of the disease. Pyrexia in suspected cases with a history of contact with abortion products or feces should raise suspicion for the disease.

## Background

Brucellosis is the most common zoonosis caused by *Brucella* infection. The disease is classified as one of the category B infectious diseases in China. According to reports, the average annual growth rate of brucellosis in 2003–2014 is 20.8%, and it will continue to rise over the next 5 years [1].

The symptoms of human brucellosis include undulant fever, weight loss, night sweats, joint pain, enlarged lymph nodes and hepatosplenomegaly. Because the clinical manifestations of brucellosis are diverse and nonspecific, a missed or incorrect diagnosis for brucellosis is possible, especially for clinically suspected cases [2–5]. Clinically suspected brucellosis cases are defined as individuals with clinical manifestations and epidemiological profiles who test positive by the plate agglutination test (PAT). In fact, clinically suspected brucellosis cases include individuals with suboptimal health, misdiagnosed brucellosis cases, and patients with other diseases [6]. The clinically suspected cases lack standardized treatment and management protocols. Some of these suspected cases may develop chronic brucellosis, which poses a serious burden for treatment [7].

In this article, we investigated the risk factors of the confirmed cases, suspected cases, and people without brucellosis to raise awareness among physicians and suspected cases.

## Methods

### Definitions

The diagnosis of brucellosis was based on the “Diagnostic criteria for brucellosis” (WS269–2007).

#### A confirmed case

A confirmed case was defined (1) by epidemiological history; (2) by characteristic clinical findings and (3) as having either positive blood cultures for *Brucella* or a serum agglutination *brucella* antibody titer of ≥1:100.

#### A suspected case

A suspected case was defined (1) by epidemiological history; (2) by characteristic clinical findings and (3) as having a standard plate agglutination titer of ≥0.04 and a serum agglutination *brucella* antibody titer of ≤1:50.

#### An asymptomatic infection

The difference between a confirmed case and a person with asymptomatic infection is that the latter was free of clinical symptoms and no organs were damaged.

Except for the suspected cases, confirmed cases and people with asymptomatic infection, the remainder of the visitors to the Songyuan CDC from 2009 to 2012 were negative for brucellosis.

### Study protocol

A self-designed questionnaire was used to collect information, including demographic characteristics (sex, age, nation, education level, and occupation), contact with animals, manner of contact and clinical symptoms (Additional file 1), and the initial visit site was the Songyuan Center for Disease Control and Prevention (CDC) from January 1st, 2009 to December 31st, 2012. We excluded those cases that had a history of brucellosis and whose questionnaire missed important information that could not be supplemented, such as the exposure history and laboratory findings. Finally, we enrolled a total 3557 people (2860 with clinical symptoms and 697 without clinical symptoms).

**Fig. 1:**
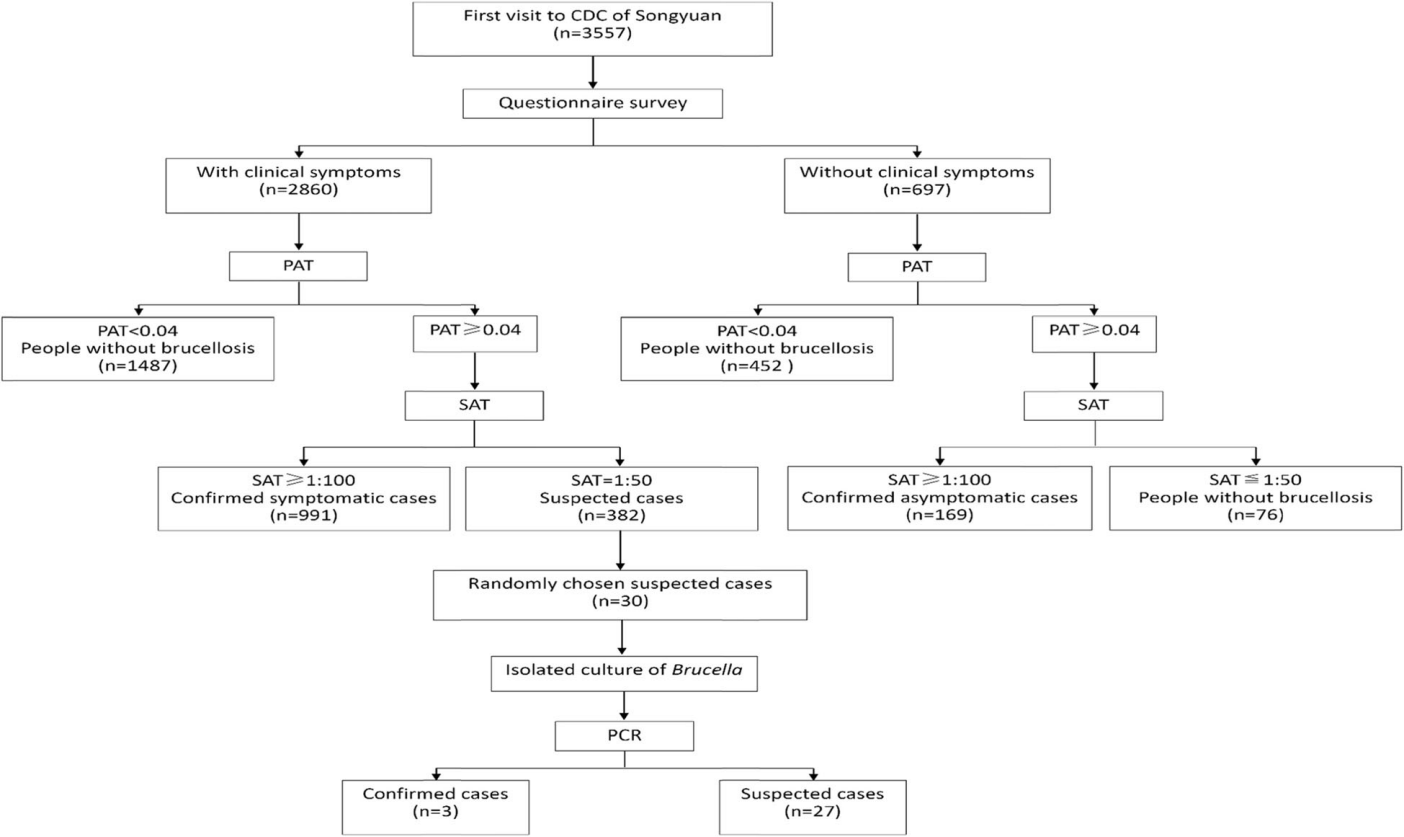
Study protocol

Blood samples were collected from all the enrollees. Based on a titer of < 0.04 detected by the plate agglutination test (PAT), we found 1939 people (1487 with clinical symptoms and 452 without clinical symptoms) without brucellosis. Based on the criteria of titer of ≥1:100 or ≤ 1:50 with the serum agglutination test (SAT), we determined 991 confirmed symptomatic cases, 382 suspected cases, 169 confirmed asymptomatic cases, and 76 people without brucellosis. Because brucellosis is characterized by the acute or insidious onset of fever and one or more symptoms, including night sweats, arthralgia, headache, fatigue, anorexia, myalgia, weight loss, arthritis/spondylitis, meningitis, or focal organ involvement (endocarditis, orchitis/epididymitis, hepatomegaly, splenomegaly), we randomly chose 30 suspected cases with fever to investigate the possibility of diagnosing brucellosis using the *Brucella* culture and validation with agarose electrophoresis or AMOS-PCR products [8] (Fig. 1). The sequence of PCR primers is listed in Additional file 2.

### Data management and analysis

Normally distributed data were displayed as the mean and standard deviation (*x* ±s). The median and Q1 to Q3 (25th to 75th percentiles, respectively) are shown. The chi-square test was used to compare the demographic characteristics, contact route, and clinical symptoms among the three groups (i.e., confirmed cases, suspected cases, and people without brucellosis). When the expected values in any of the cells of a contingency table were below 1 or more than 20% of the cells had an expected count less than 5, Fisher’s exact test was used. The difference revealed by the chi-square test was further analyzed using the Bonferroni adjustment method. The adjusted significance level α was 0.017, and statistical significance was attained when a *p*-value was less than this value. Multinomial logistic regression was used to confirm factors influencing the occurrence of brucellosis. The assignment of independent variables is shown in Table 1. For the brucellosis risk factors, we chose *P* < 0.05 for the inclusion criteria, 0.05 ≤ *P* ≤ 0.10 for the suspected risk criteria, and *P* > 0.1 for the exclusion criteria. The data were calculated using Epi-Data version 3.1 software and STATA version 12.6.

**Table 1:** Assignment of independent variables

Variable	Assignment
Sex	Men = 1, Women = 2
Age	66~86 years old = 1, 56~65 years old =2, 46~55 years old =3, 36~45 years old =4, 26~35 years old =5, 14~25 years old =6, 1~13 years old = 7
Education	Primary = 1, Junior = 2, Senior =3, Undergraduate and above =4, Unknown =5, Illiteracy =6
Occupation	Farmer and herdsmen =1, Unknown = 2, Nonfarmer and nonherdsmen =3
Contact with abortion	Yes = 1, No = 2
Contact with fur	Yes = 1, No = 2
Contact with feces	Yes = 1, No = 2
Contact with dust	Yes = 1, No = 2
Family member of infected	Yes = 1, No = 2

## Results

### Baseline characteristics

From 2009 to 2012, a total 3557 individuals were enrolled in this study and further divided into three groups (confirmed cases: 991; suspected cases: 382; people without brucellosis: 2015). We compared the differences among the three groups using the chi-square test and found significant differences in demographic characteristics, contact history (except with deer, canine, dairy and meat), and clinical symptoms (except dizziness, cough, wrist pain, omalgia, sacroiliac pain, and lymphatic swelling) (*P* < 0.05) (Tables 2, 3 and 4).

**Table 2:** Demographic characteristics of the participants

	Confirmed case n (%)		Suspected case n (%)		People without brucellosis n (%)		C vs S vs Pe			C vs S			C vs Pe			S vs Pe		
							*X*^*2*^		*P*	*X*^*2*^		*P*	*X*^*2*^		*P*	*X*^*2*^		*P*
Sex							89	.141	< 0.001	3	.877	0.049	82	.664	< 0.001	18	.482	< 0.001
Men	759	(76.59)	273	(71.47)	1205	(59.80)												
Women	232	(23.41)	109	(28.53)	810	(40.20)												
Age							64	.861	< 0.001	10	.32	0.112	36	.423	< 0.001	34	.573	< 0.001
≤ 13	21	(2.12)	6	(1.57)	132	(6.55)												
14~25	99	(9.99)	24	(6.28)	234	(11.61)												
26~35	162	(16.35)	69	(18.06)	364	(18.06)												
36~45	281	(28.36)	128	(33.51)	524	(26.00)												
46~55	271	(27.35)	92	(24.08)	470	(23.33)												
56~65	129	(13.02)	56	(14.66)	225	(11.17)												
≥ 66	28	(2.83)	7	(1.83)	66	(3.28)												
Education							21	.509	0.018	5	.452	0.340	18	.901	0.002	3	.947	0.557
Illiteracy	52	(5.25)	15	(3.93)	116	(5.76)												
Primary edu	611	(61.65)	234	(61.26)	1202	(59.65)												
Junior edu	296	(29.87)	112	(29.32)	559	(27.74)												
Senior edu	27	(2.72)	17	(4.45)	99	(4.91)												
Undergraduate & above	3	(0.30)	3	(0.79)	29	(1.44)												
Unknown	2	(0.20)	1	(0.26)	10	(0.50)												
Occupation							155	.747	< 0.001	12	.754	0.415	123	.857	< 0.001	54	.654	< 0.001
Farmers and herdsmen	838	(84.56)	331	(86.65)	1527	(75.78)												
Livestock merchant	6	(0.61)	3	(0.79)	21	(1.04)												
Livestock slaughterer	9	(0.91)	1	(0.26)	14	(0.69)												
Dairy processor	6	(0.61)	2	(0.52)	7	(0.35)												
Fur-making worker	2	(0.20)	0	(0.00)	2	(0.10)												
Other worker	10	(1.01)	4	(1.05)	60	(2.98)												
Veterinarian	3	(0.30)	2	(0.52)	7	(0.35)												
Doctor or nurse	0	(0.00)	1	(0.26)	6	(0.30)												
Student	13	(1.31)	7	(1.83)	75	(3.72)												
Children	12	(1.21)	2	(0.52)	79	(3.92)												
Unemployed	3	(0.30)	0	(0.00)	35	(1.74)												
Officer	0	(0.00)	2	(0.52)	30	(1.49)												
Freelancer	25	(2.52)	7	(1.83)	99	(4.91)												
Unknown	64	(6.46)	20	(5.24)	53	(2.63)

**Table 3:** Contact history of the participants

	Confirmed case n (%)		Suspected case n (%)		People without brucellosis n (%)		C vs S vs Pe		C vs S		C vs Pe		S vs Pe	
							*X*^*2*^	*P*	*X*^*2*^	*P*	*X*^*2*^	*P*	*X*^*2*^	*P*
Contact with animals														
Cattle							12.891	0.002	2.486	0.115	5.338	0.021	9.601	0.002
yes	85	(8.58)	23	(6.02)	228	(11.32)								
no	906	(91.42)	359	(93.98)	1787	(88.68)								
Sheep							65.415	< 0.001	1.217	0.270	58.904	< 0.001	17.383	< 0.001
yes	654	(65.99)	240	(62.83)	1032	(51.22)								
no	337	(34.01)	142	(37.17)	983	(48.78)								
Swine							9.649	0.008	9.742	0.002	2.532	0.112	4.785	0.029
yes	64	(6.46)	44	(11.52)	163	(8.09)								
no	927	(93.54)	338	(88.48)	1852	(91.91)								
Deer							0.582	0.843	–	–	–	–	–	–
yes	5	(0.50)	1	(0.26)	7	(0.35)								
no	986	(99.50)	381	(99.74)	2008	(99.65)								
Canine							2.376	0.305	–	–	–	–	–	–
yes	162	(16.35)	59	(15.45)	287	(14.24)								
no	829	(83.65)	323	(84.55)	1728	(85.76)								
Contact with substances														
Abortion products							213.426	< 0.001	1.945	0.163	194.577	< 0.001	72.951	< 0.001
yes	446	(45.01)	156	(40.84)	414	(20.55)								
no	545	(54.99)	226	(59.16)	1601	(79.45)								
Dairy and meat							4.182	0.124	–	–	–	–	–	–
yes	34	(3.43)	15	(3.93)	101	(5.01)								
no	957	(96.57)	367	(96.07)	1914	((94.99)								
Fur							43.090	< 0.001	0.011	0.915	35.375	< 0.001	16.015	< 0.001
yes	649	(65.49)	249	(65.18)	1090	(54.09)								
no	342	(34.51)	133	(34.82)	925	(45.91)								
Feces							84.272	< 0.001	0.370	0.543	66.699	< 0.001	45.770	< 0.001
yes	231	(23.31)	95	(24.87)	238	(11.81)								
no	760	(76.69)	287	(75.13)	1777	(88.19)								
Dust							15.424	< 0.001	2.490	0.115	15.426	< 0.001	0.977	0.323
yes	305	(30.78)	101	(26.44)	485	(24.07)								
no	686	(69.22)	281	(73.56)	1530	(75.93)

**Table 4:** Clinical symptoms of the participants

	Confirmed case n (%)		Suspected case n (%)		People without brucellosis n (%)		C vs S vs Pe			C vs S		C vs Pe		S vs Pe	
							*X*^*2*^		*P*	*X*^*2*^	*P*	*X*^*2*^	*P*	*X*^*2*^	*P*
Fever							330	.505	< 0.001	8.466	0.004	300.494	< 0.001	86.074	< 0.001
yes	732	(73.86)	252	(65.97)	811	(40.25)									
no	259	(26.14)	130	(34.03)	1204	(59.75)									
Chills							10	.363	0.006	0.081	0.776	9.314	0.002	3.409	0.065
yes	117	(11.81)	43	(11.26)	168	(8.34)									
no	874	(88.19)	339	(88.74)	1847	(91.66)									
Acratia							7	.303	0.026	0.812	0.367	7.235	0.007	0.794	0.373
yes	392	(39.56)	141	(36.91)	696	(34.54)									
no	599	(60.44)	241	(63.09)	1319	(65.46)									
Hyperhidrosis							119	.031	< 0.001	3.369	0.066	113.247	< 0.001	30.548	< 0.001
yes	369	(37.24)	122	(31.94)	389	(19.31)									
no	622	(62.76)	260	(68.06)	1626	(80.69)									
Dizziness							0	.490	0.783	–	–	–	–	–	–
yes	21	(2.12)	8	(2.09)	50	(2.48)									
no	970	(97.88)	374	(97.91)	1965	(97.52)									
Headache							57	.392	< 0.001	0.222	0.638	51.125	< 0.001	21.371	< 0.001
yes	235	(23.71)	86	(22.51)	269	(13.35)									
no	756	(76.29)	296	(77.49)	1746	(86.65)									
Cough							0	.153	0.926	–	–	–	–	–	–
yes	20	(2.02)	8	(2.09)	45	(2.23)									
no	971	(97.98)	374	(97.91)	1970	(97.77)									
Joint and muscle pain							63	.716	< 0.001	0.158	0.691	55.357	< 0.001	23.535	< 0.001
yes	359	(36.23)	134	(35.08)	470	(23.33)									
no	632	(63.77)	248	(64.92)	1545	(76.67)									
Muscular soreness							24	.294	< 0.001	0.387	0.534	17.532	< 0.001	14.175	< 0.001
yes	150	(15.14)	63	(16.49)	200	(9.93)									
no	841	(84.86)	319	(83.51)	1815	(90.07)									
Omalgia							2	.676	0.262	–	–	–	–	–	–
yes	43	(4.34)	16	(4.19)	65	(3.23)									
no	948	(95.66)	366	(95.81)	1950	(96.77)									
Wrist pain							0	.582	0.837	–	–	–	–	–	–
yes	5	(0.50)	1	(0.26)	7	(0.35)									
no	986	(99.50)	381	(99.74)	2008	(99.65)									
Lumbago							41	.410	< 0.001	2.899	0.089	41.003	< 0.001	6.558	0.010
yes	198	(19.98)	61	(15.97)	228	(11.32)									
no	793	(80.02)	321	(84.03)	1787	(88.68)									
Coxalgia							19	.362	< 0.001	0.092	0.762	15.361	< 0.001	10.847	0.001
yes	63	(6.36)	26	(6.81)	66	(3.28)									
no	928	(93.64)	356	(93.19)	1949	(96.72)									
Sacroiliac pain							0	.925	0.738	–	–	–	–	–	–
yes	3	(0.30)	2	(0.52)	6	(0.30)									
no	988	(99.70)	380	(99.48)	2009	(99.70)									
Gonalgia							10	.661	0.005	0.748	0.387	6.065	0.014	7.560	0.006
yes	140	(14.13)	61	(15.97)	222	(11.02)									
no	851	(85.87)	321	(84.03)	1793	(88.98)									
Dolor vagus							27	.616	< 0.001	0.143	0.705	21.253	< 0.001	13.960	< 0.001
yes	224	(22.60)	90	(23.56)	317	(15.73)									
no	767	(77.40)	292	(76.44)	1698	(84.27)									
Lymphatic swelling							4	.580	0.079	–	–	–	–	–	–
yes	2	(0.20)	3	(0.79)	3	(0.15)									
no	989	(99.80)	379	(99.21)	2012	(99.85)									
Enlargement of testis							21	.422	< 0.001	0.232	0.630	20.433	< 0.001	7.883	0.005
yes	42	(4.24)	14	(3.66)	31	(1.54)									
no	949	(95.76)	368	(96.34)	1984	(98.46)									

*C* Confirmed case; S Suspected case; *Pe* People without brucellosis

Primary edu: primary education; Junior edu: Junior middle school education; Senior edu: Senior middle school education

We further investigated the difference between any two groups using the Bonferroni adjustment (*P* < 0.017). For the demographic characteristics, our results revealed no difference between the confirmed and suspected cases. However, the confirmed cases were significantly different from the people without brucellosis, and the suspected cases exhibited significant differences from the people without brucellosis (except in education, *P* = 0.557) (Table 2).

After comparing the history of contact with animals between the confirmed and suspected cases, we found that only contact with swine was a significant characteristic (*P* = 0.002). Nevertheless, contact with swine showed no difference between the confirmed cases and the people without brucellosis (*P* = 0.012) or between the suspected cases and the people without brucellosis (*P* = 0.029). For contact with sheep, significant differences existed between the confirmed cases and the people without brucellosis and between the suspected cases and the people without brucellosis (all *P* < 0.001). Moreover, for contact with cattle, the people without brucellosis showed no difference with the confirmed cases (*P* = 0.021) but exhibited a significant difference with the suspected cases (*P* = 0.002). In addition, with respect to contact with substances, our results indicated no difference between the confirmed and suspected cases, but both the confirmed and suspected cases exhibited significant differences with the people without brucellosis (except for contact with dust in the suspected cases vs the people without brucellosis, *P* = 0.323) (Table 3).

When comparing the clinical symptoms, we found no difference between the confirmed and suspected cases (except with fever, *P* = 0.004). However, the confirmed cases were significantly different from the people without brucellosis (*P* = 0.065), and the suspected cases exhibited significant differences from the people without brucellosis (*P* = 0.373) (except with chills and acratia) (Table 4).

### Analysis of risk factors of brucellosis

We used multivariable logistic regression to identify the risk factors for brucellosis, and our results demonstrated that sex (adjusted odds ratio [aOR]: 2.249; 95% confidence interval [CI]: 1.864–2.712), age (14~86 years old) (aOR: 2.186; 95% CI: 1.037–4.608), occupation (farmers and herdsmen, and unspecified occupation) (aOR: 1.434; 95% CI: 1.052–1.953 and aOR: 5.071; 95% CI: 3.091–8.319, respectively), and contact with abortion products (aOR: 2.513; 95% CI: 2.040–3.096) were significantly associated with the risk of brucellosis in the confirmed cases (all *P* < 0.05). In addition, sex (aOR: 1.652; 95% CI: 1.284–2.126), occupation (farmers and herdsmen; unspecified occupation) (aOR: 1.895; 95% CI: 1.195–3.004 and aOR: 4.646; 95% CI: 2.341–9.220, respectively), contact with abortion products (aOR: 2.186; 95% CI: 1.037–4.608), and contact with feces (aOR: 1.506; 95% CI: 1.039–2.183) were significantly associated with the risk of brucellosis in the suspected cases (all *P* < 0.05) (Table 5).

**Table 5:** Influencing factors of brucellosis by multinomial logistic regression. Reference: people without brucellosis; sex of control: women; age of control: 1 to 13 years old; profession of control: nonfarmer and nonherdsmen; contact history of control: no contact

Group	Influencing factor	B		Std. Error		Wald		Sig.		Exp(B)		95% CI for Exp(B)			
												Lower		Upper	
Confirmed case	Sex	0	.810	0	.096	71	.773	0	.000	2	.249	1	.864	2	.712
	Age														
	14~25-years-old	0	.652	0	.327	3	.966	0	.046	1	.920	1	.010	3	.647
	26~35-years-old	0	.674	0	.323	4	.351	0	.037	1	.963	1	.042	3	.700
	36~45-years-old	0	.790	0	.316	6	.269	0	.012	2	.204	1	.187	4	.091
	46~55-years-old	0	.889	0	.315	7	.966	0	.005	2	.432	1	.312	4	.507
	56~65-years-old	0	.917	0	.325	7	.947	0	.005	2	.501	1	.322	4	.730
	66~86-years-old	0	.782	0	.381	4	.224	0	.040	2	.186	1	.037	4	.608
	Farmers and herdsmen	0	.360	0	.158	5	.218	0	.022	1	.434	1	.052	1	.953
	Unspecified occupation	1	.624	0	.253	41	.333	0	.000	5	.071	3	.091	8	.319
	Abortion products	0	.922	0	.106	75	.005	0	.000	2	.513	2	.040	3	.096
Suspected case	Sex	0	.502	0	.129	15	.240	0	.000	1	.652	1	.284	2	.126
	Farmers and herdsmen	0	.639	0	.235	7	.388	0	.007	1	.895	1	.195	3	.004
	Unspecified occupation	1	.536	0	.350	19	.288	0	.000	4	.646	2	.341	9	.220
	Abortion products	0	.714	0	.144	24	.629	0	.000	2	.043	1	.541	2	.708
	Feces	0	.409	0	.189	4	.670	0	.031	1	.506	1	.039	2	.183

### Germ culture of blood samples

**Fig. 2:**
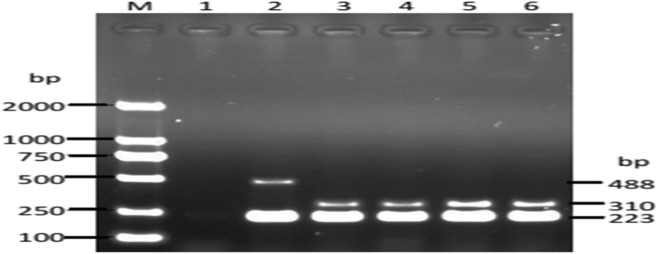
Agarose electrophoresis of AMOS-PCR products. PCR amplification using B4/B5, AMOS-M-F, AMOS-A-F, AMOS-R primers identify 3 brucella strains. M: DNA markers; 1: Negative control; 2: 544A; 3: 16 M; 4–6: Isolated strains

To investigate the possibility of diagnosing brucellosis in the suspected cases, we randomly chose blood samples from 30 suspected cases with fever to culture *Brucella* and found the bacterium in the blood samples of three cases. Furthermore, we used AMOS-PCR and agarose electrophoresis to identify the detailed species of the *Brucella* strains and found the species to be *Brucella melitensis* (Fig. 2).

## Discussion

Brucellosis is a common zoonotic infection caused by *Brucella*. This bacterial disease has not only a considerable influence on human and animal health but also a major socioeconomic impact because of loss in husbandry [9, 10]. Each year, there are 5,000,000 to 6,000,000 brucellosis patients and 500,000 new cases worldwide [11]. Furthermore, most cases of brucellosis are underdiagnosed and underreported because of vague flu-like symptoms, nonstandard medications, and difficulty in diagnosis [12].

Routine laboratory tests for the diagnosis of brucellosis include the PAT and SAT. If PAT and SAT identify suspected cases and trigger physicians to pay close attention to persistent symptoms in these suspected cases, *Brucella* culture from blood samples is a further determining method. Using the *Brucella* culture, Basappa G. Mantur found that 7.14% of suspected cases had brucellosis with negative PAT and SAT results [6]. Over 30% of clinically suspected cases are confirmed by this method to have brucellosis [13]. Similarly, we used this method and found that 10% of clinically suspected cases had brucellosis. However, this method is still deficient in providing information on the *Brucella* strains [14, 15]. Thus, we used AMOS-PCR to further determine the *Brucella* strains. Interestingly, the only strain we identified was *Brucella melitensis*. Because sheep-raising is the main economic source for farmers and herdsmen in Songyuan, sheep are one of the animals with which they most frequently come into contact. Moreover, *Brucella melitensis* (sheep) has much higher pathogenicity for humans than *Brucella abortus* (cattle) or *Brucella suis* (pig) [16]_._ These reasons at least partly support our findings. For these reasons, *Brucella melitensis* is the most prevalent *Brucella* species in Songyuan, Jilin province [17].

A high incidence of brucellosis exists in this province. As an agro-pastoral region, sheep, cattle, swine, deer, and canine are the main livestock. There is no clear border between the feeding areas and living areas. Moreover, the livestock waste is not subjected to sanitary treatment [18]. Contact with livestock and substances are the main activities contributing to brucellosis in Songyuan. We investigated the contribution of contact with livestock (sheep, cattle, swine, deer, and canine) and contact with substances (abortion products, dairy and meat, fur, feces, and dust) to the brucellosis risk. Our results revealed that abortion products were a risk factor for brucellosis both in the confirmed and suspected cases. Notably, feces were a risk factor for brucellosis only in the suspected cases. This is probably because the excreta eliminated by livestock such as cattle and sheep into the litter or into the air is not treated in time, and the *Brucella* in the feces enters the air to form infectious aerosol particles which infect humans through the respiratory system. Studies have shown that dust in sand and air may carry *Brucella* and can be transmitted by inhalation of infectious aerosol particles [19]. Unlike contact with abortion products, contact with feces is an indirect method for the confirmed and suspected cases, and infection of the respiratory tract by *Brucella* is the main route of fecal transmission. However, we could not provide the follow-up findings and repetition results of serology in the suspected cases with respiratory findings. Additionally, physicians pay more attention to suspected cases and possibly inquire further about contact information from these cases. These reasons may provide an explanation for feces being a risk factor for brucellosis in the suspected cases.

During the epidemiological investigation in Songyuan, we found that sex, farmers, and herdsmen were also risk factors for brucellosis in both the confirmed and suspected cases. Interestingly, constituent ratios exhibited an increasing tendency from the confirmed cases to the suspected cases to the people without brucellosis, in student, officers and people with senior middle school education or undergraduate and above education (Table 2).

After comparing the clinical symptoms, we found that the constituent ratio of the pyrexia cases exhibited a decreasing tendency from the confirmed cases to the suspected cases to the people without brucellosis (Table 4). Because the constituent ratio of pyrexia in the suspected cases was significantly higher than that in the people without brucellosis (*P* < 0.001), physicians should pay much more attention to pyrexia in suspected cases.

The authors, Basappa G. Mantur [6], and Wand Yi [13] performed retrospective studies and found cases misdiagnosed with brucellosis. Intriguingly, Catherine Kansiime [20] performed a prospective study and found that 31.8% of suspected cases ultimately develop brucellosis. These results further confirm that suspected cases remain at risk of brucellosis.

This study has limitations. We found that age (14–86) was a risk factor for brucellosis in the confirmed cases but not in the suspected cases. We cannot provide a sufficient explanation for this discrepancy, which merits further study in the future.

## Conclusion

Abortion products and feces are the main risk factors for brucellosis in confirmed and suspected cases, and feces was a risk factor for brucellosis only in the suspected cases. This study confirms the need for policy makers to educate farmers about health care, avoiding unprotected contact with animal abortion products or feces, and wearing masks as often as possible. In addition, pyrexia in suspected cases with a history of contact with abortion products and feces should raise suspicion for the disease. The authors suggest further investigation of the main route of fecal transmission in suspected cases.

## Supplementary information

**Additional file 1.** Case questionnaire – for outpatient of brucellosis.

**Additional file 2.** Oligonucleotide sequences of primers used in AMOS-PCR.

## Data Availability

The datasets used and/or analyzed during the current study are available from the corresponding author on reasonable request.

## Abbreviations

CDC: Center for Disease Control and Prevention
PAT: Plate agglutination test
SAT: Serum agglutination test
