# Supplementary material for: Associated factors in distinguishing patients with brucellosis from suspected cases
Source: BMC Infect Dis. 2019 Dec 9;19:1038. doi: 10.1186/s12879-019-4662-3 (PMC6902461; doi:10.1186/s12879-019-4662-3)
Supplement: Supplementary file 1 — Additional file 1. Case questionnaire – for outpatient of brucellosis. [file 12879_2019_4662_MOESM1_ESM.docx]

Case questionnaire – for outpatient of brucellosis

-Instructions-

The purpose of the case questionnaire is to investigate risk factors for brucellosis in Songyuan, Jilin province. Please answer each question.

-Questions-

1. General situation

1.1 Name: 1.2 Sex:

1.3 Nationality: 1.4 Date of birth: / /

1.5 family address:

1.6 Contact mobile: home:

1.7 Education level

□Illiteracy □Primary □Junior □Senior □Undergraduate and above

1.8 Occupation

□Famers and herdsmen □Livestock merchant □Livestock slaughter

□Dairy processor □Fur making worker □Doctor or nurse □Veterinarian

□Student □Children □Unemployed □Officer □Freelancer

Or others

2. The incidence and treatment status

2.1 Whether it is acute phase of brucellosis? □Yes □No

2.2 Date of onset: / /

2.3 Initial symptoms of brucellosis:

2.4 The time of diagnosis: / /

2.5 Do you have treatment before diagnosis of brucellosis?

□No

□Yes The last time for treatment: / /

The duration of treatment:

Medical institution:

Diagnostic test results:

Did you take medicine in the past? □Yes □No

What kind of medication did you take?

3. Anamnesis

□Rheumatic fever □Rheumatic arthritis □Pulmonary tuberculosis (TB)

□Typhoid or paratyphoid fever □Other

4. Contact history

4.1 Contact animal

□Cattle □Sheep □Swine □Deer □Canine □Other _

4.2 Contact manner

□Abortion □Dairy and meat □Gut □Fur □Soil □Feces □Dust

4.3 Contact route

□Skin and mucosa □Digestive tract □Respiratory tract □Other _

4.4 Family member of infection

□Father □Mother □Children □Husband □Wife □Other _

5. Diagnostic test results

5.1 Clinical diagnosis

5.1.1 The result of routine blood tests:

The result of ultrasonic testing

□Hepatomegaly □Splenomegaly □Other _

5.1.2 Symptoms and signs

□Fever duration: _ Body temperature: _

□Lymphatic swelling □Enlargement of testis □Joint and muscle pain

□Acratia □Hyperhidrosis □Chill

Other symptoms:

□Dizzy □Cough □Headache □Omalgia □Wrist pain

□Lumbago □Coxalgia □Sacroiliac pain □Gonalgia □Dolor vagus □Anorexia □Mental Retardation □Other _

5.1.3 Clinical stage

□Acute □Sub-acute □Chronic □Residual

5.2 Laboratory examination

5.2.1 Plate agglutination test (PAT) □- □＋ titer:

5.2.2 Serum agglutination test (SAT) titer:

5.2.3 Isolated culture of *Brucella* □- □＋

5.2.4 Polymerase chain reaction (PCR) □- □＋

The species of Brucella strain:

5.2.5 Other laboratory tests

Rose-Bengal Plate Agglutination Test:

Skin Allergy Test:

Complement Fixation Test:

Coombs Test: titer:

Enzyme-linked Immune Sorbent Assay:

5.3 Therapeutic regimen

5.3.1 Western medicine

□Streptomycin and tetracycline antibiotics

□Rifampicin and doxycycline

□Azithromycin and Rocephin

□Other _

5.3.2 Traditional Chinese medicine (TCM)**:** _

5.3.3 Other treatment methods: _

5.4 Outcome

□ [Cure](http://dict.bioon.com/detail.asp?id=bcab451354) □[Improved](http://dict.bioon.com/detail.asp?id=131f451369) □Uncrude
