# Supplementary material for: Associated factors in distinguishing patients with brucellosis from suspected cases
Source: BMC Infect Dis. 2019 Dec 9;19:1038. doi: 10.1186/s12879-019-4662-3 (PMC6902461; doi:10.1186/s12879-019-4662-3)
Supplement: Supplementary file 2 — Additional file 2. Oligonucleotide sequences of primers used in AMOS-PCR. [file 12879_2019_4662_MOESM2_ESM.docx]

**Oligonucleotide sequences of primers used in AMOS-PCR**

| **Primer pairs** | | **Nucleotide sequences (5′-3′)** | **Product size (bp)** |
| --- | --- | --- | --- |
| B4/B5 | B4 | TGGCTCGGTTGCCAATATCAA | 223 |
|  | B5 | CGCGCTTGCCTTTCAGGTCTG |  |
| BMAS | BMAS-M-F | TTATCGCTGTCACTGTTGC | 310 |
|  | BMAS-A-F | CAAGGCTTTCGCTCCATCA | 488 |
|  | BMAS-R | ATCTCAAGGCAACGGCTC |  |
